# A brain-inspired ISMO-PNN framework for neurally-grounded bearing fault diagnosis

**DOI:** 10.3389/fnbot.2026.1807995

**Published:** 2026-03-20

**Authors:** Fan Zhang, Bangcheng Zhang

**Affiliations:** 1 School of Mechatronic Engineering, Changchun University of Technology, Changchun, China; 2 Faculty of Mechanical and Electrical Engineering, Changchun Institute of Technology, Changchun, China

**Keywords:** bearing health monitoring, brain-inspired computation, improved spider monkey optimization, neurally-grounded fault diagnosis, probabilistic neural network, robotic systems

## Abstract

**Introduction:**

In advanced robot systems, monitoring the health of key components such as bearings in the transmission system is crucial for achieving reliable autonomous operation. However, there are still challenges in accurately diagnosing bearing faults under dynamic and noisy conditions.

**Methods:**

To address this issue, this paper propose a brain-inspired computational framework that integrates an Improved Spider Monkey Optimization algorithm with a Probabilistic Neural Network (ISMO-PNN) for neurally-grounded bearing fault diagnosis in robotic systems. The main content includes: (1) extracting a 22 dimensional mixed feature set from vibration signals, (2) using intelligent PCA strategy to reduce the dimensionality of features to three dimensions while retaining more than 80% of the discriminative information, and (3) using ISMO algorithm to automatically optimize the key smoothing parameters of PNN.

**Results:**

On the CWRU bearing dataset, the ISMO-PNN model has a fault classification accuracy of 97.14% and a macro-average F1 score of 97.32%, which is superior to other comparative models in the article. In addition, the minimum training and testing accuracy difference of the model is 0.72%, indicating strong generalization ability.

**Discussion:**

This brain-inspired framework, synergizing a neurally-grounded probabilistic classifier with a bio-inspired swarm optimizer, forms a robust and efficient embedded health monitoring model, which can provide feasible solutions for the development of advanced robot systems.

## 1 Introduction

In recent years, robot technology has gradually evolved from structured industrial environments to dynamic, unstructured services and collaborative scenarios, which have put unprecedented demands on the reliability and safety of robot drive systems (Rehman et al., 2025). As the core motion execution components of these systems, the health status of the drive trains is a direct determinant of motion accuracy, task performance, and overall operational safety. As critical components within the drive train, bearings are one of the key components that are most prone to failure. Therefore, accurate and robust early fault detection of bearings is an important measure for implementing predictive maintenance protocols and minimizing unplanned catastrophic downtime (Li et al., 2025; Li et al., 2025).

Traditional fault diagnosis techniques for bearings, such as spectral analysis and time-domain statistics based on vibration signals, have shown good performance in controlled laboratory environments. These methods have clear physical interpretability and low computational costs, laying the foundation for health status monitoring (Smith and Randall, 2015). However, in the working environment of real machines, which often involve complex tasks like motion control, obstacle avoidance, and multi-agent collaboration (Sun et al., 2025; Xie et al., 2025; Liu et al., 2025), vibration signals often degrade due to complex background noise, variable load conditions, and non-stationary operating states, which limits the generalization performance and diagnostic reliability of traditional methods (Zhang et al., 2025; Li et al., 2025; Li et al., 2025). Their diagnostic accuracy often declines significantly under variable speeds and loads, which are common in robotic operations (Chen et al., 2024). Recent advanced diagnostic models, such as those employing deep learning and adaptive signal processing, have been developed to enhance robustness against noise and variable conditions (Che et al., 2020; Sohaib et al., 2017). These methods demonstrate superior capability in handling non-stationary signals compared to traditional techniques. Therefore, developing adaptive and highly robust intelligent diagnostic algorithms that can manage uncertainty is crucial for maintaining the long-term autonomous and safe operation of advanced robot systems (Gao et al., 2024).

In recent years, in order to address these challenges, some scholars have proposed computational methods inspired by the information processing mechanisms of the biological nervous system (Mishra and Behera, 2025). The Probabilistic Neural Network (PNN) classification model based on Bayesian decision theory has attracted widespread attention in the industry due to its close relationship with the decision-making process of biological nervous systems under uncertainty and its ability to support the development of interpretable diagnostic models (Liu and Cai, 2025). PNN is particularly noted for its fast-training speed and inherent probabilistic output, which provides a measure of classification confidence (Specht, 1990). The integration of advanced signal processing with PNN has shown promising results in improving diagnostic accuracy. Related neural-dynamics approaches have also shown effectiveness in solving robot motion planning and control problems under noisy conditions (Sun et al., 2023; Lian et al., 2024).

However, the performance of PNN largely depends on its core smoothing parameters (spread). The suboptimal selection of this parameter will greatly undermine classification accuracy and generalization ability. Manually tuning this parameter is inefficient and often fails to achieve optimal performance across diverse fault conditions. Swarm intelligence optimization algorithms, including the Spider Monkey Optimization (SMO) algorithm proposed by Bansal et al., simulate the cooperative foraging behavior of animal populations (Bansal et al., 2014). These algorithms have significant potential in solving complex optimization problems and can automatically and efficiently adjust PNN parameters (Rajeshwari and Sughasiny, 2023). Nevertheless, the standard SMO algorithm may suffer from premature convergence, indicating room for improvement in balancing global and local search. This limitation can prevent it from finding the globally optimal parameters for complex models like PNN when applied to high-dimensional or noisy feature spaces.

Building upon these insights, this paper proposes a brain-inspired ISMO-PNN framework for neurally-grounded bearing fault diagnosis in robotic systems. To ensure practicality in resource-aware applications, the model employs Principal Component Analysis (PCA) (Liu et al., 2023) to reduce the dimensionality of mixed features. An improved spider monkey optimization (ISMO) algorithm is then applied to automatically optimize the smoothing factor of the probabilistic neural network (PNN), thereby addressing its parameter sensitivity. The effectiveness and advantages of the proposed ISMO-PNN framework are validated through experiments on a public bearing dataset. Its structure will be detailed in subsequent chapters.

The remaining structure of this article is as follows: Section 2 elaborates on the basic methods and principles of the model proposed in this article; Section 3 introduces the experimental setup and conducted a comprehensive analysis of the results based on the public CWRU bearing dataset; Section 4 summarizes this study and discusses possible future research directions.

## 2 Methodology

This section proposes a fault diagnosis model for robot drive system bearings that integrates feature engineering, dimensionality reduction, and meta heuristic optimization probability modeling. Firstly, discriminative features are automatically extracted from noisy vibration signals. Then, PCA is used to compress the feature space to improve computational efficiency. Finally, the improved Spider Monkey Optimization (ISMO) algorithm is combined with PNN to form **t**he proposed brain-inspired ISMO-PNN framework. This model aims to achieve high diagnostic accuracy and strong generalization ability while maintaining the practicality of embedded health status monitoring. The following sections provide a detailed introduction to each component stage: experimental data and preprocessing, feature extraction and dimensionality reduction, ISMO-PNN diagnostic model, as well as experimental setup and evaluation metrics.

### 2.1 Experimental data and preprocessing

The study employs the widely recognized benchmark dataset for rotating machinery fault diagnosis, made publicly available by the Case Western Reserve University (CWRU) Bearing Data Center (Smith and Randall, 2015). This dataset includes vibration signals captured at various sampling frequencies; this paper selected data sampled at 12 kHz, collected by an accelerometer attached to the motor’s drive end.

**Figure 1:**
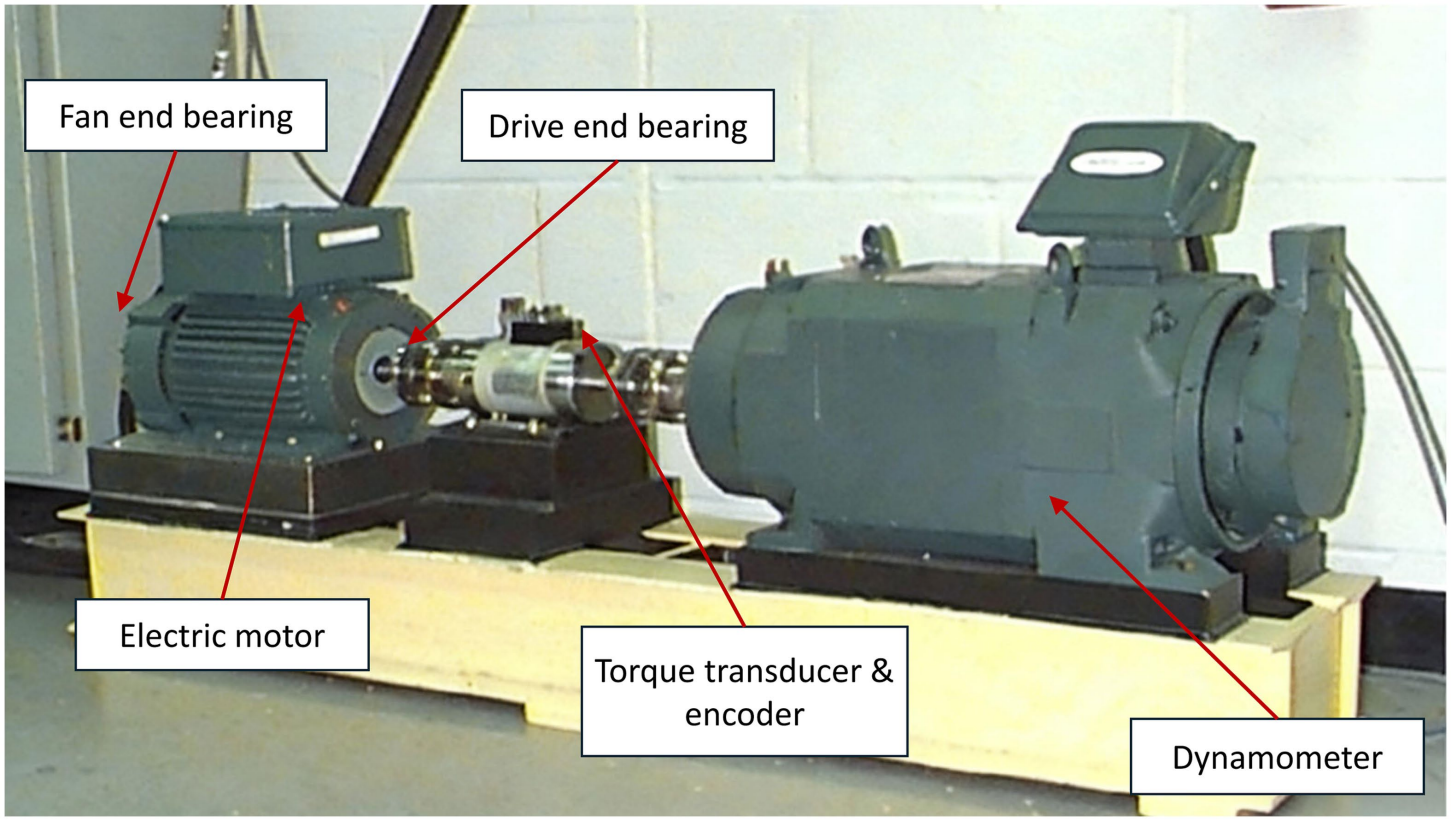
Schematic of the CWRU bearing test rig.

The experimental setup, whose schematic is presented in Figure 1, was configured to simulate four primary bearing health states: Normal, Ball Fault, Inner Race Fault, and Outer Race Fault. These faults were artificially induced using electro-discharge machining, with fault diameters ranging from 0.007 to 0.040 inches, and data were recorded across different motor load conditions (0 to 3 horsepower).

To prepare the data for supervised learning, this article implemented the following preprocessing steps:

#### 2.1.1 Data segmentation

This article divided continuous vibration signals into fixed-length samples, setting the sample length L to 1,024 data points and applying a 50% overlap between consecutive segments. The choice of 1,024 points was made to ensure that each sample contains sufficient vibration information (approximately 0.085 s at 12 kHz sampling rate) for effective feature extraction, while maintaining computational efficiency suitable for embedded monitoring applications. The 50% overlap strategy was adopted to augment the training dataset and improve model robustness, as it helps capture fault-related patterns that may appear at different phases of the signal while preserving sufficient independence between adjacent samples. This approach not only maximizes the utilization of available data but also maintains sufficient sample independence, ultimately yielding 17,503 valid samples for further analysis.

#### 2.1.2 Dataset partitioning

All generated samples were randomly divided into three distinct subsets: training, validation, and test. This article used a 7:2:1 split ratio, resulting in 12,252 training samples, 3,500 validation samples, and 1,751 test samples. This random partitioning was executed carefully to ensure that all fault classes were represented proportionally in each subset, thus avoiding potential bias.

#### 2.1.3 Data standardization

To facilitate model training and improve convergence, this study normalized both the raw vibration signals (prior to feature extraction) and the subsequently extracted features. This article employed *Z*-score standardization, computed using the statistics of the training set to prevent data leakage. The standardization formula is defined as Equation 1:


$$z=\frac{x-\mu }{\delta }$$ (1)

Where x is the original value, and μ and δ are the mean and standard deviation calculated from the training set, respectively.

### 2.2 Feature extraction and dimensionality reduction

#### 2.2.1 Feature extraction from multiple domains

To comprehensively characterize bearing vibration signals under different health states, this article extracted 22 features covering four analysis domains: time, frequency, demodulation (envelope), and nonlinear analysis. A complete list of these features, including their definitions and physical interpretations, is provided in Appendix A. The detailed classification of the feature set is as follows:

*Time-Domain Statistical Features (Features 1–10)*: This category encompasses common metrics such as mean, standard deviation, root mean square (RMS), peak and peak-to-peak values, skewness, kurtosis, shape factor, impulse factor, and margin factor. These features directly quantify the signal’s amplitude distribution, energy content, and shock intensity, rendering them highly sensitive to the early onset of fault development (Zhang et al., 2024).

*Envelope Spectrum Features (Features 11–13)*: First, this article obtained the envelope signal by applying the Hilbert Transform to the raw vibration data. From the spectrum of this envelope signal, then extracted three key features: spectral peak magnitude, its corresponding frequency, and spectral mean. The envelope spectrum excels at isolating periodic impact components caused by localized bearing defects, making these features critical for diagnosing specific fault types such as ball and inner race faults (Zou et al., 2024).

*Frequency-Domain Statistical Features (Features 14–21)*: Include spectral centroid, spectral RMS, spectral variance, spectral skewness, spectral kurtosis, spectral peak frequency, spectral peak magnitude, and Spectral Entropy (Chen et al., 2024). Spectral Entropy quantifies the uncertainty or randomness in the distribution of spectral energy, providing a measure of the signal’s complexity in the frequency domain as Equation 2:


$$H_{\text{spec}}=-\sum_{k=1}^{K}p_{k}\log_{2}(p_{k})$$ (2)

Where $p_{k}=(M[k]/\sum_{i=1}^{K}M[i])$ is the normalized spectral magnitude (a scalar) at frequency bin *k*, and M[k] is the spectral magnitude at frequency bin k.

(4) *Non-Linear Feature (Feature 22)*: Wavelet Entropy. A 3-level decomposition using the “db4” wavelet was performed. The Shannon entropy of the energy distribution across the detail coefficients captures transient impacts and non-linear dynamic characteristics as Equation 3:


$$H_{\text{wavelet}}=-\sum_{j=1}^{L}{\tilde{p}}_{j}\log_{2}({\tilde{p}}_{j})$$ (3)

Where ${\tilde{p}}_{j}=(E_{j}/\sum_{i=1}^{L}E_{i})$ is the normalized energy (a scalar)at the j-thdecomposition leveland $E_{j}$ is the energy of the wavelet coefficients at the j-th decomposition level.

#### 2.2.2 Feature dimensionality reduction *via* principal component analysis (PCA)

Using all 22 features directly can lead to information redundancy, high computational cost, and the “curse of dimensionality.” Therefore, Principal Component Analysis (PCA) was employed for unsupervised dimensionality reduction (Hotelling, 1933). PCA transforms the original features into a new set of orthogonal axes (principal components) ordered by the amount of variance they explain.

Given the standardized training set feature matrix $X\in {\mathbb{R}}^{m\times 22}$ (where m is the number of samples), its covariance matrix is $C=(1/(m-1))X^{T}X$, X is a matrix, and C is a 22 × 22 covariance matrix. Eigenvalue decomposition is performed on C as Equation 4:


$$Cv_{i}=\lambda_{i}v_{i}$$ (4)

Where $\lambda_{i}$ is the i-th eigenvalue and $v_{i}$ is its corresponding eigenvector (the direction of the i-th principal component).

The variance contribution ratio of each principal component is $\left(\lambda_{i}/\sum \lambda_{i}\right)$. A cumulative variance contribution threshold of 80% was set. Then selected the smallest number of components, k, that satisfies as Equation 5:


$$\frac{\sum_{i=1}^{k}\lambda_{i}}{\sum_{j=1}^{22}\lambda_{j}}\geq 0.8$$ (5)

Experiments determined k=3, the feature dimensionality was reduced from 22 to 3. This process retained 80.53% of the discriminative information from the original data while reducing the total data volume by 86.4%, significantly improving computational efficiency for the subsequent model.

### 2.3 The ISMO-PNN diagnostic model

#### 2.3.1 Fundamentals of probabilistic neural network (PNN)

The Probabilistic Neural Network (PNN) is a feedforward network based on Bayesian decision theory and Parzen window probability density estimation (Specht, 1990). It serves as a neurally-grounded probabilistic classifier for decision-making under uncertainty. For an input vector $x\in {\mathbb{R}}^{d}$ (here d=3) to be classified, the PNN computes the posterior probability that it belongs to class $\theta_{i}$. Its decision function $D_{i}(x)$ is defined as Equation 6:


$$D_{i}(x)=\frac{1}{\left(2\pi^{d/2}\sigma^{d}N_{i}\right)}\sum_{k=1}^{N_{i}}\exp(-\frac{x-{x_{\mathrm{ik}}}^{2}}{2\sigma^{2}})$$ (6)

Where $N_{i}$ is the number of training samples belonging to class $\theta_{i}$; $x_{\mathrm{ik}}$ is the k-th training sample from class $\theta_{i}$; σ is the smoothing parameter (Spread), the single critical hyperparameter of the PNN. It controls the width of the Gaussian kernel, directly influencing the smoothness of the classification boundary and the model’s generalization ability.

The final decision rule of the PNN is to assign the input x to the class $\theta_{i}$ with the largest $D_{i}(x)$ value.

#### 2.3.2 Improved spider monkey optimization (ISMO) algorithm

The Spider Monkey Optimization (SMO) algorithm is a meta-heuristic that mimics the fission-fusion social foraging behavior of spider monkeys (Bansal et al., 2014). This work introduces an Improved SMO (ISMO) as a bio-inspired swarm intelligence optimizer to automatically tune the PNN’s key parameter. The standard SMO can converge prematurely. This article introduced two key improvements to form the ISMO algorithm for optimizing the PNN’s *σ* parameter.

1. Non-linear Adaptive Perturbation Rate (PR) (Agrawal et al., 2018): The fixed or linearly changing PR was modified to decay non-linearly with the iteration count t as Equation 7:

$$\mathrm{PR}(t)={\mathrm{PR}}_{\max}-({\mathrm{PR}}_{\max}-{\mathrm{PR}}_{\min})\times {\left(\frac{t}{T_{\max}}\right)}^{\alpha }$$ (7)

Where $T_{\max}$ is the maximum number of iterations, and α>1 (here set α=2). A high PR value in early iterations promotes global exploration, while a low value in later iterations favors local exploitation, achieving a better search balance.

1. Elite-guided Local Search: During the local leader phase, the position update incorporates both the global best solution and the local leader information to enhance exploitation capability. Specifically, with probability $P_{\text{elite}}$, the update mechanism follows Equation 8, where the global best solution $G_{\text{best}}$ guides the search direction while maintaining diversity through the local leader term (Swami et al., 2017) as Equation 8:

$$\begin{array}{l} X_{\mathrm{new}}(j)=X(j)+\mathrm{rand}()\cdot (G_{\text{best}}-X(j))\\ +\text{randn}()\cdot (\mathrm{LL}(j)-X(j)) \end{array}$$ (8)

Where X(j) is the current position of the j-th spider monkey in the population, LL(j) is the position of the local leader for the group to which the j-th spider monkey belongs and rand() denotes a random number uniformly generated in the range [0, 1].

The paper defines the fitness function of the ISMO algorithm as the minimization of the average classification error rate obtained through 5-fold cross-validation of the PNN model on the training set. The optimization variable is the smoothing parameter *σ*, with a search space of [0.01, 5]. Then, the optimal σ value determined by the ISMO program is used to configure and construct the final PNN diagnostic model.

#### 2.3.3 Implementation steps of ISMO-PNN framework

The steps of the proposed ISMO-PNN framework are as follows:

*Step 1*: Data preprocessing and feature extraction.

The original vibration signal is first segmented into fixed length samples with overlap. Extract a comprehensive set of 22 statistical, spectral, and nonlinear features from each sample to form the initial high-dimensional set.

*Step 2*: Standardize and split the dataset.

Normalize the extracted features using Z-score normalization, with parameters calculated from the training set. Then, the entire dataset is randomly divided into training, validation, and testing subsets according to a preset ratio.

*Step 3*: Perform feature dimensionality reduction through PCA.

PCA is applied to standardize training features. The number of principal components is automatically selected to preserve over 80% of the original variance, significantly reducing the computational burden of feature dimensions and subsequent steps.

*Step 4*: Hyperparameter optimization based on ISMO.

Use ISMO algorithm to search for the optimal smoothing parameter (*σ*) of PNN. This algorithm iteratively evaluates candidate parameters by training a PNN model and performs 5-fold cross validation on the training set to evaluate its performance.

*Step 5*: Final model training and evaluation.

Train the final PNN model on the entire reduced training set using the optimal σ * found by ISMO, and evaluate the accuracy, precision, recall, and F1 score of the model.

### 2.4 Experimental setup and evaluation metrics

All experiments were conducted in a consistent computing environment using MATLAB R2024a for data processing, feature extraction, statistical analysis, and model implementation. The Statistics and Machine Learning Toolbox was utilized for implementing benchmark classifiers, while custom code was developed for the proposed ISMO-PNN framework. The ISMO parameters were set as: population size N=20, maximum iterations $T_{\max}=20$, ${\mathrm{PR}}_{\max}=0.9$, ${\mathrm{PR}}_{\min}=0.1$, and elite guidance probability $P_{\text{elite}}=0.3$.

To comprehensively evaluate the model performance, this paper adopted the following evaluation metrics: (1) Accuracy, which defines the proportion of correctly classified test samples. (2) Precision, recall, and F1 score for each fault category, which are used to evaluate the precision and recall rates of classification in detail. (3) Macro-average F1 score (the arithmetic mean of the F1 score for each category), which provides a robust and balanced measure of overall model performance in multi-class classification. (4) Confusion matrix, which is used to visualize the classification results and examine the distribution of errors across all health states.

## 3 Experiments and results analysis

This section conducts a comprehensive evaluation of our proposed ISMO-PNN model using the public CWRU bearing dataset (Smith and Randall, 2015). Firstly, the overall diagnostic performance of ISMO-PNN is benchmarked against several other models. Secondly, this article rigorously evaluates the inherent generalization ability and operational reliability of the ISMO-PNN algorithm. All evaluations are based on the consistent data partitioning and feature engineering process described in Section 2.

### 3.1 Comparative analysis of comprehensive diagnostic performance

This article compared the ISMO-PNN model against five representative machine learning benchmarks. These include: the original Probabilistic Neural Network (PNN) with its default smoothing parameter (*σ* = 0.1); a Support Vector Machine with a Radial Basis Function kernel (SVM-RBF) (Cortes and Vapnik, 1995); a K-Nearest Neighbors (KNN) classifier (Cover and Hart, 1967); a Decision Tree model (Loh, 2011); and a Random Forest ensemble (Breiman, 2001). For a fair comparison, all models were trained and tuned (where applicable) on the same training set and their final performance was evaluated on the identical, held-out test set.

**Figure 2:**
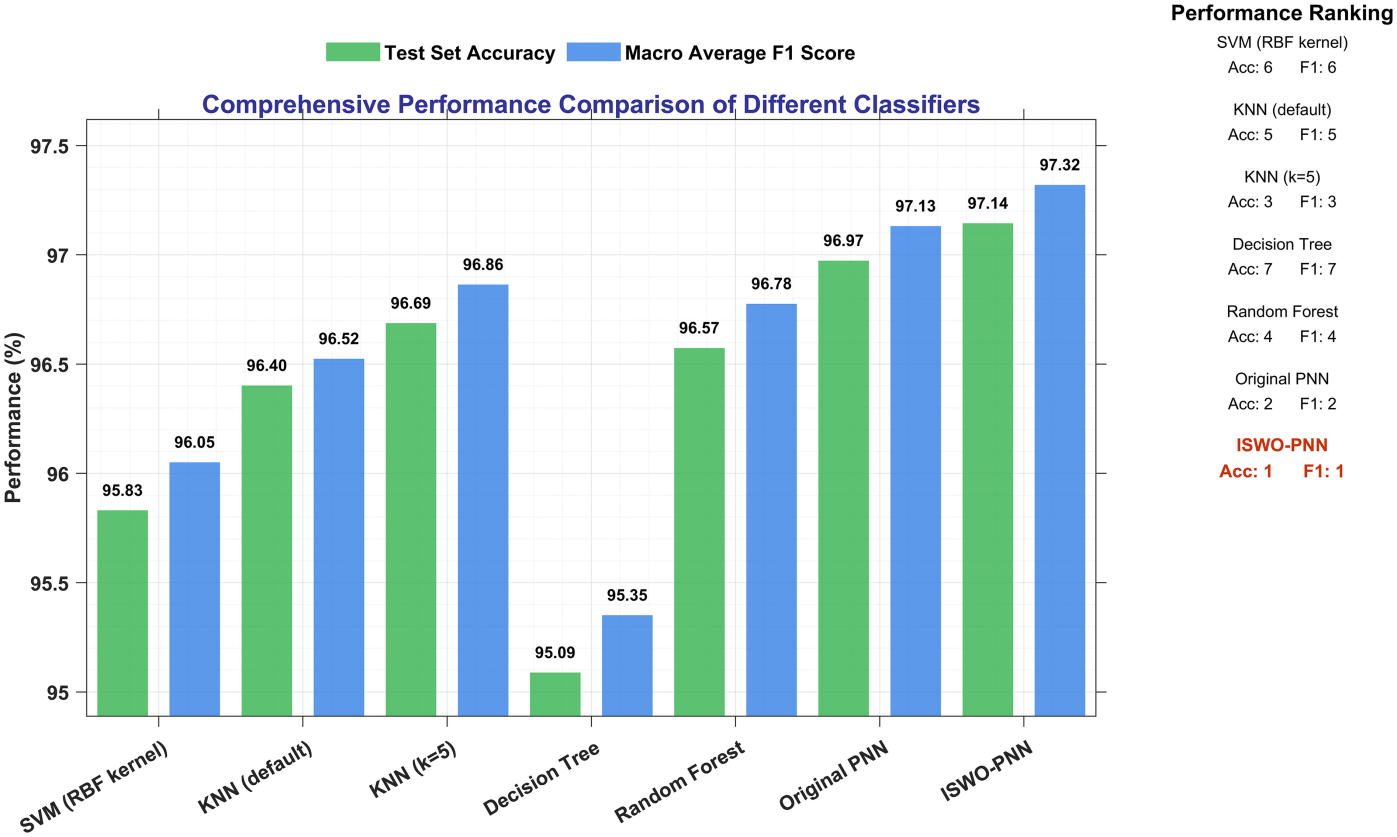
Enhanced comprehensive performance.

The key performance metrics on the test set are summarized in Table 1 and Figure 2. Our ISMO-PNN model achieved the highest scores in both overall classification accuracy (97.14%) and macro-average F1-score (97.32%). When compared to the original PNN with its default parameter, ISMO-PNN shows modest but clear improvements of 0.17 and 0.19 percentage points in accuracy and F1-score, respectively. Despite the small numerical value, this continuous gain verifies the effectiveness of using our improved Spider Monkey optimization algorithm to fine-tune the key smoothing parameters of PNN, making the model better align with the underlying feature distribution of the data.

**Table 1:** Comprehensive performance comparison of different models on the test set.

Model	Accuracy (%)	Macro-avg F1-score (%)
SVM (RBF Kernel)	95.83	96.05
KNN	96.40	96.52
Decision tree	95.09	95.35
Random forest	96.57	96.78
Original PNN(*σ* = 0.1)	96.97	97.13
**ISMO-PNN**	**97.14**	**97.32**

Bold values represents the method adopted in this paper.

From the comparison results between the ISMO-PNN model and other models, it can be observed that the SVM-RBF model performs relatively poorly, primarily because we used the default kernel parameters without fine-tuning, which fails to adapt to the specific data characteristics. Both the KNN and decision tree models exhibited a significant decrease in accuracy when transitioning from the training set to the test set, indicating a potential risk of data overfitting. In contrast, the PNN model, with its inherent regularization governed by the smoothing parameter *σ*, demonstrates more stable generalization capabilities. The random forest ensemble method achieved an accuracy of 96.57%, which is the closest to our model. However, ISMO-PNN offers practical advantages in terms of computational complexity and model footprint, making it particularly significant for embedded applications.

### 3.2 Detailed performance and robustness analysis of the ISMO-PNN model

#### 3.2.1 Convergence analysis and optimal parameter determination

**Figure 3:**
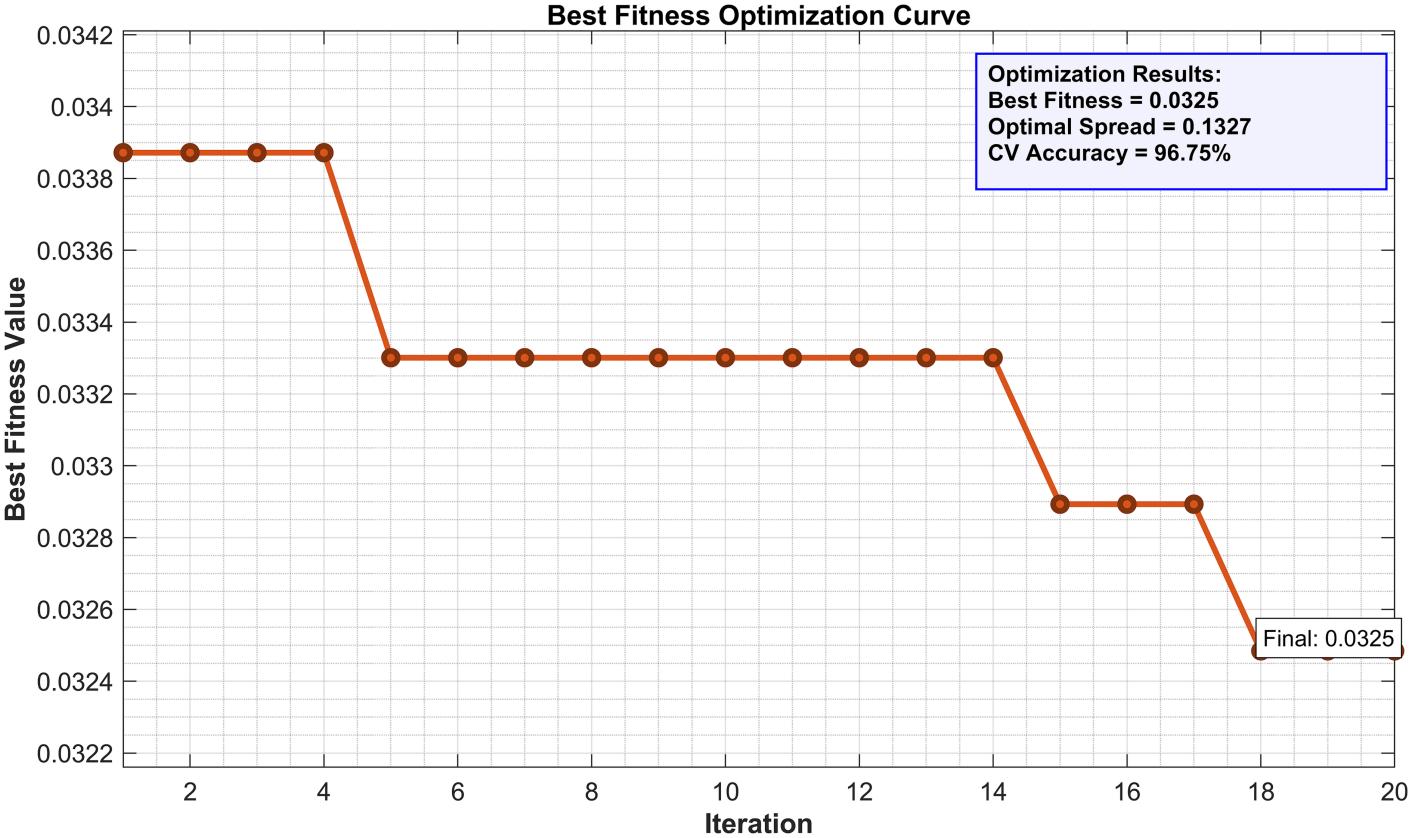
Fitness optimization curve of the ISMO algorithm.

Figure 3 shows the fitness optimization curve of ISMO during 20 iterations, depicting the process of the algorithm gradually converging to the optimal solution. After searching, the fitness value obtained is 0.0325, corresponding to an average 5-fold cross validation accuracy of 96.75%. Unlike the commonly used default value of 0.1, the optimal smoothing parameter determined by the algorithm is *σ* * = 0.1327, providing direct evidence for the necessity and value of automated and data-driven parameter optimization strategies in this diagnostic task.

#### 3.2.2 Per-class diagnostic performance and error analysis

The performance under each health condition is listed in Table 2. It can be seen that the ISMO-PNN model has an accuracy and recall of 100% under “normal” conditions, and its performance is relatively balanced in the three fault states, with F1 scores exceeding 95.4% for each fault category. Among them, the category of “Inner Race Fault” achieved the highest recall rate (97.88%), indicating that the ISMO-PNN model has a stronger ability to detect this fault, while the accuracy of the category of “Inner Race Fault” was the highest (96.24%), indicating that the model has the lowest false alarm rate for this category. Overall, the model has reliability and sensitivity to different faults.

**Table 2:** Per-class performance of the ISMO-PNN model on the test set.

Class	Precision (%)	Recall (%)	F1-score (%)
Normal	100.00	100.00	100.00
Ball fault	96.24	94.71	95.47
Inner race fault	95.85	97.88	96.86
Outer race fault	96.98	96.69	96.83

**Figure 4:**
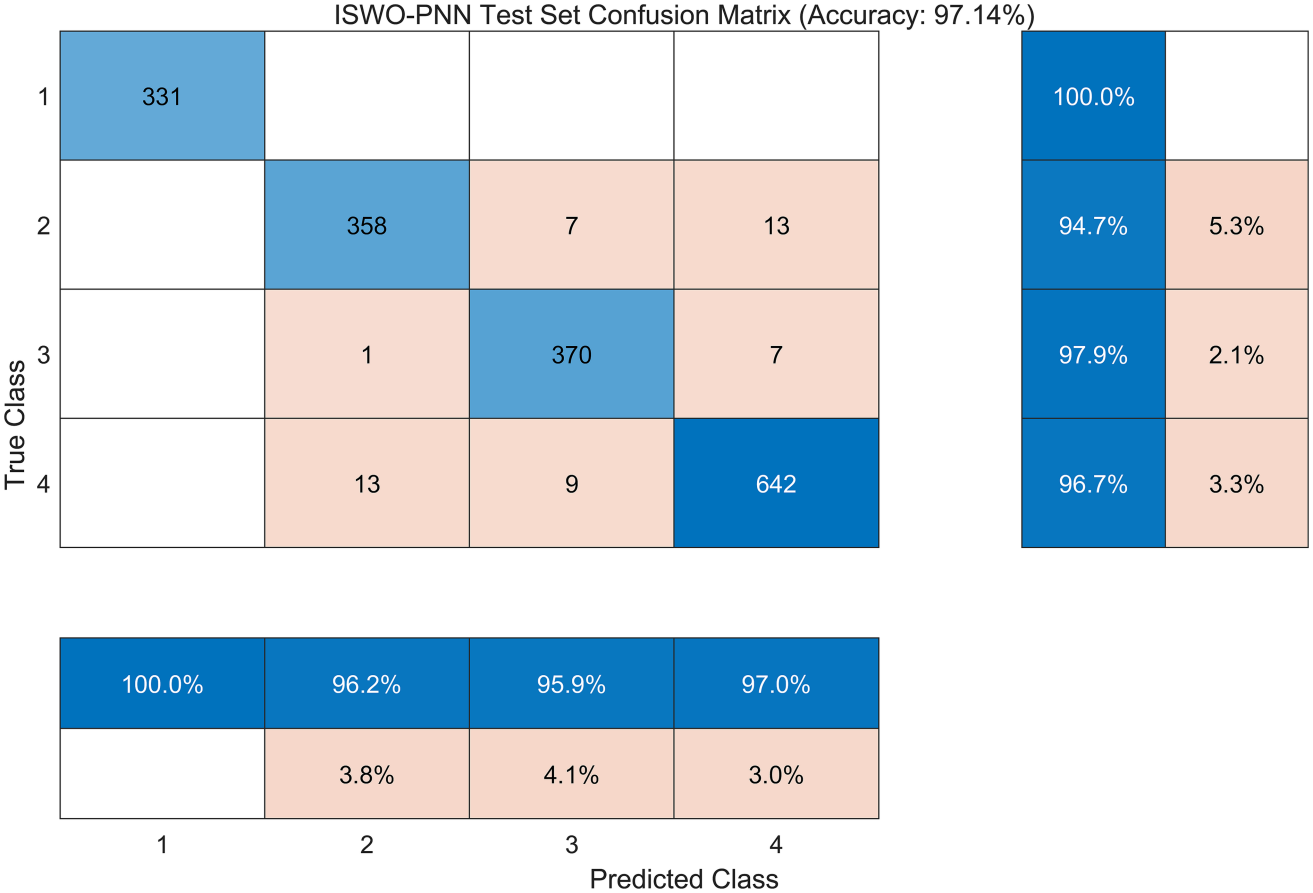
Confusion matrix of ISMO-PNN on the test set.

The confusion matrix results are shown in Figure 4. It can be seen from the figure that the ISMO-PNN model achieves completely correct classification of normal state samples. A small number of classification errors mainly focus on mutual misjudgment between three types of faults. It should be noted that in the model proposed in this article, the occurrence of fault samples being misjudged as normal is extremely rare, ensuring high reliability of fault detection (i.e., low false alarm rate). Although in a few cases, the specific types of faults may not be accurately distinguished (allowing for a certain false alarm rate), this is more meaningful from an engineering application perspective because this error pattern is more acceptable and is a more desirable trade-off in actual operation and maintenance.

#### 3.2.3 Generalization capability and feature engineering analysis

The generalization capability of the model was evaluated by its performance difference between the training set and the test set. As shown in Table 3, the train-test accuracy gap for ISMO-PNN was only 0.72%, which is significantly smaller than the 1.41% gap for the original PNN. This smaller performance degradation indicates that the optimized parameter σ* = 0.1327 helped the model achieve a better balance between memorizing the training data and maintaining generalization ability.

**Table 3:** Generalization gap (%).

Model	Training accuracy (%)	Test accuracy (%)	Accuracy gap (%)
Original PNN(σ = 0.1)	98.38	96.97	1.41
ISMO-PNN (σ* = 0.1327)	**97.86**	**97.14**	**0.72**

Bold values represents the method adopted in this paper.

**Figure 5:**
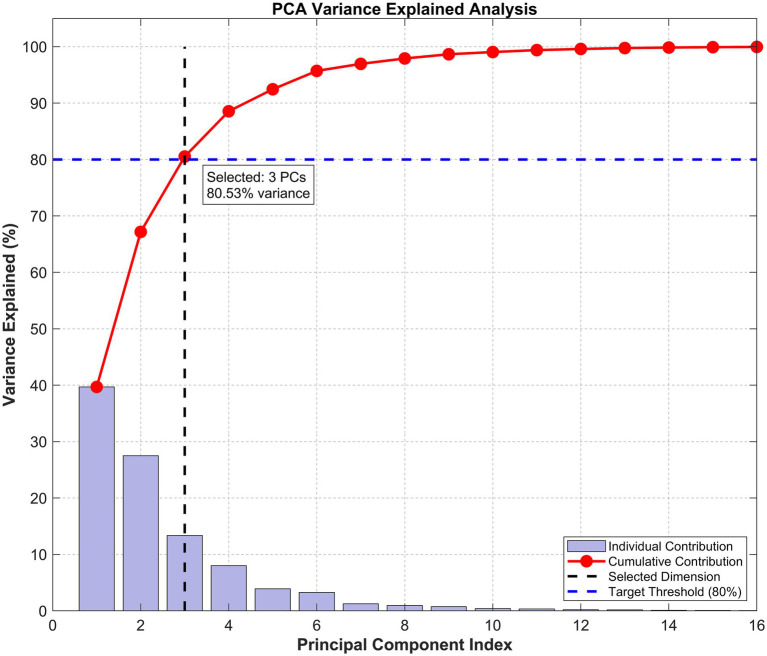
PCA dimensionality reduction variance explained analysis.

The design of the feature processing pipeline also enhanced the model’s practicality. Figure 5 illustrates the variance explained by each principal component in the PCA. From Figure 5, it can be seen that using only the first three principal components can cumulatively explain over 80% of the data variance. Therefore, this paper ultimately chose to compress the features from 22 dimensions to 3 dimensions. Table 4 shows the results of the features before and after this dimensionality reduction.

**Table 4:** Quantitative analysis of PCA dimensionality reduction effect.

Metric	Original features	Reduced features	Change
Feature dimension	22	3	−86.4%
Total data volume	385,066 points	52,653 points	−86.4%
Variance retention	100%	80.53%	−19.47%
Expected speedup	Baseline	~7×	+600%

As shown in Table 4, by using dimensionality reduction methods, the total data volume was reduced by 86.4% while retaining 80.53% of the discriminative information. This measure not only significantly reduces the computational complexity of the subsequent fault diagnosis ISMO-PNN model, but also enhances the robustness of features to noise by focusing on the main changing patterns. This also balances information retention and computational efficiency, and has practical significance for diagnostic algorithms for embedded robots.

#### 3.2.4 Reproducibility verification

To verify the stability of the method, it is necessary to study its reproducibility. This article retrained the model on the same dataset using the optimal smoothing parameter *σ* * = 0.1327 determined by ISMO. The accuracy of the newly trained model is 97.14%, and other parameters and results are completely consistent with our previous experimental results. Therefore, this precise replication under fixed random seeds proves the stability and program repeatability of our proposed ISMO-PNN model.

#### 3.2.5 Analysis

The above experimental results validate the effectiveness of integrating Bayesian theory based probabilistic neural networks with swarm intelligence based optimizers. The proposed brain-inspired ISMO-PNN framework demonstrates that the synergy between a neurally-grounded probabilistic classifier and a bio-inspired swarm optimizer can significantly enhance diagnostic robustness. The introduction of the ISMO algorithm enables automatic adjustment of key smoothing parameters in PNN, significantly enhancing the model’s fit to specific data patterns inherent in bearing fault diagnosis tasks. This collaborative effect is crucial in the design of adaptive and reflective perception modules for robot systems.

From a brain-inspired computing perspective, the combination of PNN and ISMO in our framework reflects a fundamental biological mechanism. The ISMO-based automated parameter tuning can be viewed as an analog of neuroplasticity. Just as synaptic strengths in biological neural networks are dynamically adjusted through learning to optimize information processing and decision-making, the ISMO algorithm iteratively tunes the PNN’s smoothing parameter to adapt to the statistical characteristics of the vibration data. This adaptive process enables the diagnostic system to ‘learn’ the optimal configuration for its current operating context, mirroring how biological systems maintain performance through continuous self-regulation. The framework thus embodies a form of adaptive homeostasis, where the model parameters are automatically calibrated to maintain diagnostic accuracy across varying fault conditions without manual intervention.

From a practical perspective, the ISMO-PNN framework offers several advantages that make it particularly suitable for real-world deployment. First, its relatively simple structure results in low computational overhead, making it well-suited for resource-constrained embedded platforms commonly used in mobile robots and autonomous systems. Second, the probabilistic output of the PNN provides not only a classification decision but also a measure of confidence, which is valuable for reliability-critical applications such as human-robot collaboration or autonomous navigation. Third, the automated parameter optimization via ISMO eliminates the need for manual tuning, facilitating adaptation to different operating conditions without expert intervention. These characteristics suggest that the proposed framework could be integrated into real-time condition monitoring systems for predictive maintenance in industrial robotics or service robots.

We acknowledge that while our proposed framework demonstrates strong diagnostic performance on a standard bearing dataset, its direct applicability to real robotic systems requires further validation. Future work should focus on implementing the ISMO-PNN framework on actual robotic hardware to evaluate its performance under these realistic constraints.

## 4 Conclusion

This article proposes a brain-inspired ISMO-PNN framework for neurally-grounded bearing fault diagnosis. The framework integrates multi-domain feature extraction, PCA-based dimensionality reduction, and ISMO-optimized probabilistic neural network classification.

The model was validated using the publicly available CWRU bearing dataset, and the results showed that the ISMO-PNN model has strong diagnostic performance, with a test set accuracy of 97.14% and a Macro-average F1 score of 97.32%. The comprehensive advantages of the proposed model were verified by comparing its parameters with the unoptimized PNN standard algorithm and several other traditional machine learning models. Key advantages include: (1) balanced and high recognition rates across all fault categories, (2) excellent generalization ability evidenced by a minimal train-test performance gap (0.72%), and (3) enhanced computational efficiency through PCA-based dimensionality reduction, which reduces data volume by 86.4% while retaining over 80% of discriminative information. These characteristics make the proposed framework particularly suitable for embedded health monitoring in advanced robotic systems. Future research needs to validate the robustness of this model in real robotic dynamic environments and explore the possibility of its deep integration with robotic control systems.

## Data Availability

The raw data supporting the conclusions of this article will be made available by the authors, without undue reservation.
