# Supplementary material for: A brain-inspired ISMO-PNN framework for neurally-grounded bearing fault diagnosis
Source: Front Neurorobot. 2026 Mar 20;20:1807995. doi: 10.3389/fnbot.2026.1807995 (PMC13047198; doi:10.3389/fnbot.2026.1807995)
Supplement: Supplementary file 1 [file Table_1.DOCX]

Appendix A. The complete definitions and physical interpretations of all features

| No. | Category | Feature Name (Symbol) | Mathematical Definition | Brief Interpretation |
| --- | --- | --- | --- | --- |
| 1 | Time-Domain | Mean (μ) | µ *=* $\frac{1}{N}\sum_{n=1}^{N} x\left[ n \right]$ | Average amplitude of the signal. |
| 2 | Time-Domain | Standard Deviation (σ) | σ *=* $\sqrt{\frac{1}{N-1}\sum_{n=1}^{N} {(x\left[ n \right]-\mu)}^{2}}$ | Dispersion of the signal around the mean. |
| 3 | Time-Domain | Root Mean Square ( $RMS$) | $RMS=\sqrt{\frac{1}{N}\sum_{n=1}^{N} x{[n]}^{2}}$ | Quadratic mean, representing signal power. |
| 4 | Time-Domain | Peak ($X_{peak}$) | $X_{peak}$*=* max($\left\Vert x \right.\left. [n] \right\Vert$) | Maximum absolute amplitude, indicating impact intensity. |
| 5 | Time-Domain | Peak-to-Peak ($X_{pp}$) | $X_{pp}$ *=* max$\left( x\left[ n] \right. \right.) -$ min$\left( x\left[ n \right] \right)$ | Total range of the signal amplitude. |
| 6 | Time-Domain | Skewness ($\Upsilon$) | $\Upsilon=\frac{\frac{1}{N}\sum_{n=1}^{N} (x\left[ n \right]-\mu)^{3}}{\sigma^{3}}$ | Asymmetry of the signal's probability distribution. |
| 7 | Time-Domain | Kurtosis (*K*) | *K=* $\frac{\frac{1}{N}\sum_{n=1}^{N} (x\left[ n \right]-\mu)^{4}}{\sigma^{4}}$ | "Tailedness" or impulsiveness of the distribution. |
| 8 | Time-Domain | Shape Factor ( $SF$) | $SF=\frac{RMS}{\frac{1}{N}\sum_{n=1}^{N} \left\Vert x \right.\left. \left[ n \right] \right\Vert}$ | Ratio of power to average rectified value. |
| 9 | Time-Domain | Impulse Factor ($IF$) | $IF=\frac{X_{peak}}{\frac{1}{N}\sum_{n=1}^{N} \left\Vert x \right.\left. \left[ n \right] \right\Vert}$ | Sensitivity to extreme impacts. |
| 10 | Time-Domain | Margin Factor ($MF$) | $MF=\frac{X_{peak}}{{(\frac{1}{N}\sum_{n=1}^{N} \sqrt{\left\Vert x \right.\left. \left[ n \right] \right\Vert})}^{2}}$ | Another indicator of peak severity relative to the overall signal. |
| 11 | Envelope Spectrum | Envelope Peak ($EP$) | $EP=max(Y_{env}\left[ k \right])$  where $y_{env}\left[ n \right]=\left\Vert\mathrm{Hilbert}\left( x\left[ n \right] \right) \right\Vert$, and  $Y_{env}\left[ k \right]$ is its FFT magnitude. | Peak amplitude in the envelope spectrum, highlighting repetitive impacts. |
| 12 | Envelope Spectrum | Envelope Peak Frequency ($EPF$) | $EPF={arg\max}_{k}(Y_{env}\left[ k \right])\cdot\frac{f_{s}}{N_{FFT}}$ | Frequency at which the envelope spectral peak occurs. |
| 13 | Envelope Spectrum | Envelope Mean ($EM$) | $EM=\frac{1}{K}\sum_{k=1}^{K} Y_{env}\left[ k \right]$ | Average level of the envelope spectrum. |
| 14 | Frequency-Domain | Spectral Centroid ( $SC$) | $SC=\frac{\sum_{k=1}^{K} f\left[ k \right]\cdot M\left[ k \right]}{\sum_{k=1}^{K} M\left[ k \right]}$  $M\left[ k \right]$: FFT magnitude at frequency bin $k.$ | "Center of mass" of the spectrum, indicating where the signal's energy is concentrated. |
| 15 | Frequency-Domain | Spectral RMS ( $SRMS$) | $SRMS=\sqrt{\frac{\sum_{k=1}^{K} (f\left[ k \right]^{2}\cdot M\left[ k \right])}{\sum_{k=1}^{K} M\left[ k \right]}}$ | Root mean square of frequencies, weighted by magnitude. |
| 16 | Frequency-Domain | Spectral Variance ( $SV$) | $SV=\frac{\sum_{k=1}^{K} ((f\left[ k \right]-SC)^{2}\cdot M\left[ k \right])}{\sum_{k=1}^{K} M\left[ k \right]}$ | Spread of the spectrum around its centroid. |
| 17 | Frequency-Domain | Spectral Skewness ($SSk$) | $SSk=\frac{\sum_{k=1}^{K} ((f\left[ k \right]-SC)^{3}\cdot M\left[ k \right])}{{SV}^{1.5}\cdot\sum_{k=1}^{K} M\left[ k \right]}$ | Asymmetry of the spectral distribution. |
| 18 | Frequency-Domain | Spectral Kurtosis ($SK$) | $SK=\frac{\sum_{k=1}^{K} ((f\left[ k \right]-SC)^{4}\cdot M\left[ k \right])}{{SV}^{2}\cdot\sum_{k=1}^{K} M\left[ k \right]}$ | Peakedness of the spectral distribution. |
| 19 | Frequency-Domain | Spectral Peak Frequency ($SPF$) | $SPF=f\left[ arg{max}_{k}\left( M\left[ k \right] \right) \right]$ | Frequency at which the raw spectrum magnitude is highest. |
| 20 | Frequency-Domain | Spectral Peak Magnitude ($SPM$) | $SPM=max\left( M\left[ k \right] \right)$ | Maximum magnitude in the raw spectrum. |
| 21 | Frequency-Domain | Spectral Entropy ($H_{spec}$​) | $H_{spec}=-\sum_{k=1}^{K} p_{k}\log_{2}\left( p_{k} \right)$  where $p_{k}=M\left[ k \right]/\sum_{i=1}^{K} M\left[ i \right]$ | Measures the complexity/unpredictability of the spectral power distribution. |
| 22 | Non-Linear | Wavelet Entropy ($H_{wavelet}$​) | $H_{wavelet}=-\sum_{j=1}^{L} \tilde{p}_{j}{\mathrm{lo}g}_{2}\left( \tilde{p}_{j} \right)$  where$\tilde{p}_{j}=E_{j}/\sum_{i=1}^{L} E_{i},\mathrm{and}E_{j} \mathrm{is} the \mathrm{energy}$ of the wavelet coefficient at decomposition level $j.$ | Measures the energy disorder across different time-frequency scales (using a 3-level 'db4' wavelet packet decomposition). |
